# Glucose Concentration Measurement by All-Grating-Based System

**DOI:** 10.3390/s23094216

**Published:** 2023-04-23

**Authors:** Hung-Chih Hsieh, Yi-Ming Lu, Ke-Cheng Huang

**Affiliations:** Department of Electro-Optical Engineering, National United University, No. 2 Lienda, Miaoli 36063, Taiwan

**Keywords:** heterodyne interferometer, moving grating, self-align sensor, glucose concentration

## Abstract

An accurate, easy setup, low-cost, and time-saving method for measuring glucose concentration was proposed. An all-grating-based glucose concentration measurement system contained moving-grating-based heterodyne interferometry and a grating-based self-align sensor. By combining the first-order diffraction lights from two separated moving gratings by a polarization beam splitter and creating S- and P-polarized light interference by an analyzer, the interference signal could be a heterodyne light source with a heterodyne frequency depending on the relative velocities of the two moving gratings. Next, a grating-based self-align sensor was used to make the optical configuration setup easy and accurate. Moreover, the sensor was deposited on GOx film to improve the measurement sensitivity and specificity for glucose. Finally, the phase change induced by the reaction of the sensor and glucose solutions was detected. The validity of this method was proved, and the measurement resolution can reach 2 mg/dL.

## 1. Introduction

Effective management of diabetes mellitus requires routine monitoring of blood glucose levels [1]. Fluctuations in blood glucose levels outside the reference range can result in severe complications such as weight changes, neurologic symptoms, seizures, coma, and even death [2]. Many factors influence blood glucose levels, such as dietary intake of carbohydrates, physical activity, insulin production and sensitivity, medications, stress, sleep, and illness. However, currently, available methods for blood glucose testing are invasive, causing discomfort for patients and incurring significant costs for the healthcare system [3]. Therefore, there is a need for reliable, comfortable, and noninvasive methods for real-time blood glucose measurement that can significantly improve treatment efficacy and facilitate the early detection of metabolic syndrome. Furthermore, the limitations of current invasive methods for blood glucose testing may lead to patients neglecting routine monitoring, resulting in poorly managed diabetes and an increased risk of complications. Noninvasive methods, therefore, have the potential to encourage patients to adhere to regular monitoring and improve their overall quality of life. Additionally, noninvasive methods may be especially beneficial for pediatric and elderly patients who may experience discomfort or difficulty with invasive methods. As such, there is a growing need for the development of reliable and comfortable noninvasive methods for blood glucose measurement to improve diabetes management and reduce the risk of severe complications.

Currently, glucose detection technology can be divided into electrochemical and optical measurement technology. The original detection technique used a glucose oxidase (GOx) electrode sensor proposed by Clark et al. [4] in 1962. For blood, glucose oxidase is specific to glucose and can reduce the interference of other substances on the measurement. The glucose concentration was measured according to the electrical changes at both ends of the electrode. This research has laid an essential foundation for other glucose measurement techniques.

Compared with the two measurement technologies, the development of electrochemical measurement technology is more general, and different ways of depositing GOx film have also been developed. Mutyala and Mathiyararsu et al. [5] used electrode materials to react with GOx and glucose. The product undergoes Direct Electron Transfer (DET), which uses electrical changes to measure the glucose concentration in the solution, and the measurement resolution can reach 90 mg/dL. Wei and Zhang et al. [6] used polydopamine, the method of embedding gold nanoparticles (polydopamine films with gold nanoparticles, AuNPs), to fix GOx on the sensor. This method can significantly increase the resolution of the sensor to 0.18 mg/dL. Liu et al. [7] deposited GOx, chitosan, and α phosphate (GOD/chitosan/α ZrP) complex on the electrode, and the measured glucose concentration sensitivity range was 4.5~144 mg/dL. Tan et al. [8] used Sol-gel, which was made by depositing GOx on a mixed film of chitosan (CS) and silica (SiO2), changing the ratio of CS to SiO_2_ and the production conditions. The best parameters were found, and the measurement range was 0.9~468 mg/dL. The above techniques used the specificity of GOx for detection. However, the disadvantage of electrochemical detection is that the reaction speed is slow, and the electrode of the electrochemical detection technique will directly contact the glucose solution, which will cause aging and change its electrical characteristics after long-term use. It is not easy to calibrate the blood glucose meter, which affects the measurement result.

Compared with the electrochemical measurement method, the optical measurement technology has a faster response time and is noncontact between the glucose solution and the instrument, which reduces the error sources. The principle is that the phase of the test light will be changed when the light penetrates or reflects. The study by Chen et al. [9] measured the refractive index of the glucose solutions by heterodyne interferometry. Chen et al. [10] used the Surface Plasmon Resonance (SPR) technique to analyze the phase of heterodyne interferometry and used the change in the refractive index of the glucose solution to measure its concentration. The resolution of this method can reach 0.327 mg/dL, but the probe is not easy to make and is expensive. The measurement methodologies proposed in the above two studies lacked specificity for glucose since GOx was not used. 

After introducing GOx, the interference of other substances on the measurement results can be reduced. In our previous study [11], we deposited the GOx on the surface of a single-mode fiber, and heterodyne interferometry was used to measure the phase change of the glucose solution under test. The measurement time can be significantly shortened to 1.3 s, the best measurement resolution can reach 0.141 mg/dL, and the fiber can be reused up to 10 times. To reduce the complexity of the optical configuration setup, we proposed an alternative method, integrating a Dove prism and precision circular heterodyne interferometry, for measuring the refractive index and concentration of sodium chloride and hydrogen peroxide solutions [12]. The phase error, refractive index error, and resolution of the concentration are approximately 0.003°, $2\times 10^{-5}$, and $1\times 10^{-3}$ M. However, there was a limitation in that the angle of light incident on the sample was determined by the inclination angle of the side slope of the Dove Prism, and this will limit the incident angle in the study. Furthermore, this limits the optimization of parameters that the sensor can adjust. On the other hand, the Electro-Optical Modulator (EOM) was used in the traditional heterodyne interferometer, and the EOM heterodyne system was expensive. Hence, finding a new cost-effective heterodyne light source is necessary for a heterodyne interferometer. 

In this study, we introduced an all-grating-based system for glucose concentration measurement. The EOM used in the traditional EOM-based heterodyne interferometer was replaced with a moving grating for generating the heterodyne light source. Compared with the EOM-based heterodyne interferometer, this method has the advantages of easy setup, cost-effectiveness, and no wavelength usage limitation. 

Our proposed system combined moving-grating-based heterodyne interferometry with a grating-based self-align sensor. The moving-grating-based heterodyne interferometer was composed of a pair of moving gratings to obtain a heterodyne light source. By passing light through these gratings and combining the two first-order diffraction lights with a polarization beam splitter, a heterodyne frequency could be obtained that depended on the relative velocity of the two moving gratings. To sense the glucose concentration, a grating-based self-align sensor was utilized, which was contained by two diffraction gratings to control and receive the test light beam. Additionally, the sensor was coated with a glucose oxidase (GOx) film to improve the measurement sensitivity and specificity for glucose. Finally, the phase change induced by the sensor and glucose solutions reaction was detected, providing a reliable and accurate measurement of glucose concentration. The validity of this method was proved, and the measurement resolution can reach 2 mg/dL. 

Compared with the electrochemical method, this method has no particular advantages in patient usage. First, the measurement resolution of the electrochemical method proposed by Wei and Zhang et al. [6] is better (0.18 mg/dL). Second, both this and the electrochemical methods are invasive for patients to prepare the testing sample. Moreover, although the glucose sensor can be reused, the cost of the overall optical system is expensive compared to the electrochemical method. Even if the measurement time is only 1.5 s to obtain the result, compared with the measurement time of the electrochemical method, the improvement in the measurement time was not particularly significant. On the other hand, although the resolution of the all-grating-based glucose concentration measurement method proposed in this study is not as good as traditional EOM-based heterodyne interferometer and electrochemical methods, it has the following advantages compared with the EOM-based heterodyne interferometer method. First, the advantage of the moving-grating-based heterodyne interferometer proposed in this method was cost-effectiveness. The cost of two transmission gratings and a motor-controlled linear stage was lower than the EOM and its driver. Second, the optical configuration of the sensor was designed as grating-based, which was easy to use and optically set up. In addition, the wavelength (632.8 nm) and power (20 mW) of the laser light source used in this study will not cause skin damage when irradiating the human body. Furthermore, in future work for optical heterodyne interferometer glucose sensing, a long-wavelength light source must be used to penetrate the skin layer to reach the vascular tissue, such as 780 nm or 940 nm. Commercial EOMs do not have good modulation efficiency in this wavelength range. Therefore, using the moving-grating-based heterodyne light source proposed by this method can avoid the wavelength limitation.

## 2. Principles

### 2.1. Moving-Grating-Based Heterodyne Light Source

**Figure 1:**
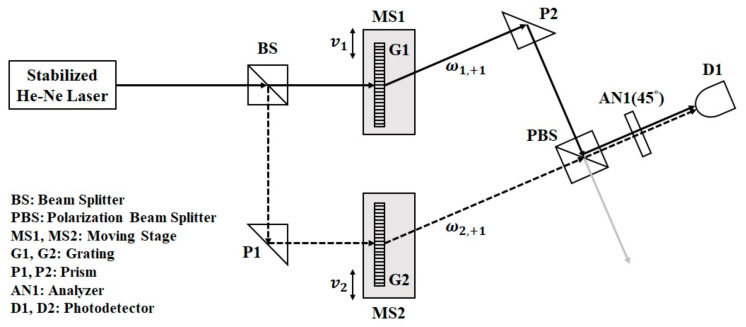
The optical configuration of the moving-grating-based heterodyne light source.

The optical configuration of the moving grating-based heterodyne light source is shown in Figure 1. For convenience, the z-axis is chosen along the light propagation direction, and the y-axis is along the vertical direction. The frequency-stabilized He–Ne laser emits linearly polarized light $E_{0}$ with a wavelength of λ and a polarization direction of 45° with respect to the x-axis divided into transmitted light $E_{t}$ (solid line) and reflected light $E_{r}$ (dotted line) through BS. These two lights will be directed into two paths: (1) the solid line: BS→G1(+1)→P2→PBS(S pol.)→AN1(45°)→D1; (2) the dotted line: BS→P1→G2(+1)→PBS(P pol.)→AN1(45°)→D1.

According to the Jones matrix calculation [13,14,15], we can calculate the electric field of the lights passing through these two paths as $E_{t,G1+1,S}$ and $E_{r,G2+1,P}$, and they can be written as

(1) $$\begin{array}{ll} E_{t,G1+1,S} & =AN1\left(45{}^{\circ}\right)\cdot {PBS\left(90{}^{\circ}\right)\cdot {G1}_{+1}\cdot BS\cdot E}_{0}\\ & =\frac{1}{2}\left(\begin{array}{cc} 1 & 1\\ 1 & 1 \end{array}\right)\cdot \left(\begin{array}{cc} 0 & 0\\ 0 & 1 \end{array}\right)\cdot \left(\begin{array}{cc} {tg}_{G1+1,P}e^{i\varphi_{G1+1,P}} & 0\\ 0 & {tg}_{G1+1,S}e^{i\varphi_{G1+1,S}} \end{array}\right)\cdot \left(\begin{array}{cc} t_{p} & 0\\ 0 & t_{s} \end{array}\right)\cdot \left(\begin{array}{c} 1\\ 1 \end{array}\right)\\ & =\frac{1}{2}\left(\begin{array}{c} t_{s}{tg}_{G1+1,S}e^{i\varphi_{G1+1,S}}\\ t_{s}{tg}_{G1+1,S}e^{i\varphi_{G1+1,S}} \end{array}\right) \end{array}$$

and

(2) $$\begin{array}{ll} E_{r,G2+1,P} & ={AN1\left(45{}^{\circ}\right)\cdot PBS\left(0{}^{\circ}\right)\cdot {G2}_{+1}\cdot BS\cdot E}_{0}\\ & =\frac{1}{2}\left(\begin{array}{cc} 1 & 1\\ 1 & 1 \end{array}\right)\cdot \left(\begin{array}{cc} 1 & 0\\ 0 & 0 \end{array}\right)\cdot \left(\begin{array}{cc} {tg}_{G2+1,P}e^{i\varphi_{G2+1,P}} & 0\\ 0 & {tg}_{G2+1,S}e^{i\varphi_{G2+1,S}} \end{array}\right)\cdot \left(\begin{array}{cc} r_{P} & 0\\ 0 & r_{s} \end{array}\right)\cdot \left(\begin{array}{c} 1\\ 1 \end{array}\right)\\ & =\frac{1}{2}\left(\begin{array}{c} t_{P}{tg}_{G1+1,P}e^{i\varphi_{G1+1,P}}\\ t_{P}{tg}_{G1+1,P}e^{i\varphi_{G1+1,P}} \end{array}\right) \end{array}$$

where $r_{P}$, $r_{S}$, $t_{p}$, and $t_{s}$ are the reflection coefficients and transmission coefficients of the S-polarization light and P-polarization light of the BS, respectively. ${tg}_{G1+1,P}$, ${tg}_{G1+1,S}$, ${tg}_{G2+1,P}$, and ${tg}_{G2+1,S}$ are the diffraction efficiency coefficients of first-order diffraction light of G1 and G2, respectively. $\varphi_{G1+1,P}$, $\varphi_{G1+1,S}$, $\varphi_{G2+1,P}$, and $\varphi_{G2+1,S}$ are the phase-shifting from the moving gratings, which are dependent on the diffraction order m, the grating pitches (*P*_1_ and *P*_2_), and the displacements ($S_{1}$ and $S_{2}$). $\varphi_{Gi}$ could be written as [16,17,18,19]

(3) $$\varphi_{Gi}=-m\cdot \frac{2\pi S_{i}}{P_{i}}\;(i=1,2\;\mathrm{and}\;m=+1)$$

According to the arrangement of the optical configuration, the interference signal detected by detector D1 is composed of the p-polarization of the positive first-order diffracted light from G2 and the s-polarization of the positive first-order diffracted light from G1. After calculating the Jones matrix, this signal can be written as

(4) $$\begin{array}{ll} I_{1}= & {\left|E_{t,G1+1,S}+E_{r,G2+1,P}\right|}^{2}\\ & =\frac{1}{2}\left(t_{S}^{2}{tg}_{G1+1,S}^{2}+r_{P}^{2}{tg}_{G2+1,P}^{2}+2t_{S}r_{P}{tg}_{G1+1,S}{tg}_{G2+1,P}\cos\left(\varphi_{G1+1,S}-\varphi_{G2+1,P}\right)\right)\\ & =\frac{1}{2}\left(I_{1DC}+I_{10}\cos\left(\varphi_{G1+1,S}-\varphi_{G2+1,P}\right)\right) \end{array}$$

where $I_{1DC}=t_{S}^{2}{tg}_{G1+1,S}^{2}+r_{P}^{2}{tg}_{G2+1,P}^{2}$ and $I_{10}=2t_{S}r_{P}{tg}_{G1+1,S}{tg}_{G2+1,P}$ are the average intensity and the amplitude of the interference signal. Since G1 and G2 are moving with velocities $v_{1}$ and $v_{2}$ by linear motorized stage, the interference signal can be rewritten as

(5) $$\begin{array}{ll} I_{1} & =\frac{1}{2}\left(I_{1DC}+I_{10}\cos\left(\varphi_{G1+1,S}-\varphi_{G2+1,P}\right)\right)\\ & =\frac{1}{2}\left(I_{1DC}+I_{10}\cos\left(2\pi \left(\frac{v_{1}}{P_{1}}-\frac{v_{2}}{P_{2}}\right)t\right)\right) \end{array}$$

From Equation (5), the phase of the interference signal oscillates in the time domain with a frequency

(6) $$f=\frac{v_{1}}{P_{1}}-\frac{v_{2}}{P_{2}}$$

This frequency is the so-called heterodyne frequency and can be used for the heterodyne light source. In this study, we used this heterodyne light source in the glucose concentration measurement system.

### 2.2. The Glucose Concentration Measurement System

Combining Equations (1) and (2) together without the AN1 is the electric field of the heterodyne light source, it can be written as

(7) $$E_{out}=\left(\begin{array}{c} r_{P}{tg}_{G2+1,P}e^{i\varphi_{G2+1,P}}\\ t_{s}{tg}_{G1+1,S}e^{i\varphi_{G1+1,S}} \end{array}\right)$$

In this study, a circular heterodyne interferometry [13] was used for the glucose concentration measurement. We simplify the configurations for convenience: (1) The BS we used in the experiment is ideal so that $r_{P}=1$ and $r_{S}=1$. (2) The identical gratings G1 and G2 are used so that $P_{1}=P_{2}=P$. (3) The diffraction efficiencies of the S-polarization of G1 and the P-polarization of G2 are identical, so that ${tg}_{G1+1,S}={tg}_{G2+1,P}=1$. (4) The moving velocities of G1 and G2 are identical in value but in opposite directions so that $v_{1}=-v_{2}=v$. Hence, the heterodyne light source could be rewritten as

(8) $${E^{\prime }}_{out}=\left(\begin{array}{c} e^{i2\pi \frac{v}{P}t}\\ e^{-i2\pi \frac{v}{P}t} \end{array}\right)$$

Let the heterodyne light source pass through a quarter-wave plate with the fast axis located in the 45° direction with respect to the x-axis. The electric field $E_{CH}$ could be written as

(9) $$\begin{array}{ll} E_{CH} & =Q\left(45{}^{\circ}\right)\cdot {E^{\prime }}_{out}=\frac{1}{2}\left(\begin{array}{c} 1\\ i \end{array}\right)e^{-i2\pi \frac{v}{P}t}+\frac{1}{2}\left(\begin{array}{c} i\\ 1 \end{array}\right)e^{i2\pi \frac{v}{P}t}\\ & =\frac{1}{2}\left(\begin{array}{c} 1\\ i \end{array}\right)e^{-i\omega t}+\frac{1}{2}\left(\begin{array}{c} i\\ 1 \end{array}\right)e^{i\omega t} \end{array}$$

where ω=2πv/P. From Equation (10), the angular frequency difference between the right- and the left-circular polarizations is 2ω.

**Figure 2:**
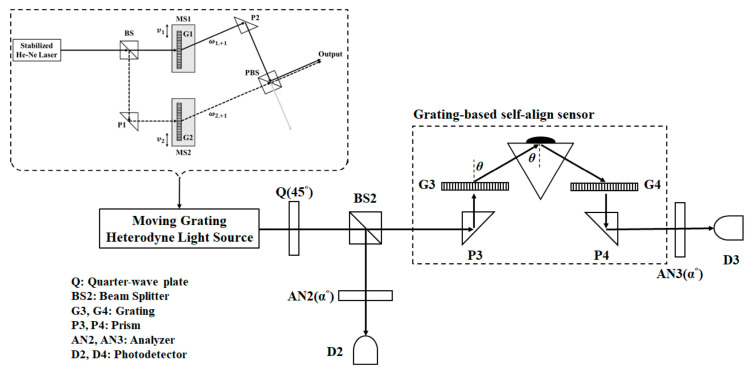
The optical configuration of glucose concentration measurement system.

The optical configuration of the glucose concentration measurement system is shown in Figure 2. The BS2 divides the circular heterodyne light source into two parts, the reflection part is used for the reference beam, and the transmission part is used for the test beam. The optical path of the reference beam is $BS2\to AN2\left(\alpha {}^{\circ}\right)\to D2$, where α is the angle of the transmission axis of the AN2. After the Jones matrix calculation, the electric field of the reference signal could be written as

(10) $$\begin{array}{ll} E_{ref} & =AN2(\alpha {}^{\circ})\cdot BS2\cdot E_{CH}\\ & =\frac{1}{2}\left(\begin{array}{cc} \cos^{2}\alpha & \sin\alpha \cos\alpha \\ \sin\alpha \cos\alpha & \sin^{2}\alpha \end{array}\right)\cdot \left(\begin{array}{cc} 1 & 0\\ 0 & 1 \end{array}\right)\cdot \left(\begin{array}{c} \cos\omega t\\ -\sin\omega t \end{array}\right)\\ & =\left(r_{Pr}\cos\alpha \cos\omega t-r_{Sr}\sin\alpha \sin\omega t\right)\left(\begin{array}{c} \cos\alpha \\ \sin\alpha \end{array}\right) \end{array}$$

The intensity of the interference signal received by D2 is

(11) $$I_{ref}={\left|E_{ref}\right|}^{2}=\frac{1}{2}\left(1+\cos\left(2\omega t+\varphi_{r}\right)\right)$$

the phase of the reference signal is $\varphi_{r}$, which is equal to 2α.

On the other hand, the optical path of the test beam is $BS2\to P3\to G3\left(+1\right)\to Sample\to G4\left(+1\right)\to P4\to AN3\left(\alpha {}^{\circ}\right)\to D3$. The test system is composed of two prisms, P3 and P4, two identical gratings, G3 and G4, and the tested sample. The purpose of the grating is to control the incident angle θ on the tested sample and to receive the reflected signal from the tested sample by the symmetric design of the test system. Hence, the electric field of the test signal could be expressed by the Jones matrix calculation:

(12) $$\begin{array}{ll} E_{test} & =AN3(\alpha {}^{\circ})\cdot R\cdot E_{CH}\\ & =\frac{1}{2}\left(\begin{array}{cc} \cos^{2}\alpha & \sin\alpha \cos\alpha \\ \sin\alpha \cos\alpha & \sin^{2}\alpha \end{array}\right)\cdot \left(\begin{array}{cc} r_{Pt} & 0\\ 0 & r_{St} \end{array}\right)\cdot \left(\begin{array}{c} \cos\omega t\\ -\sin\omega t \end{array}\right)\\ & =\left(r_{Pt}\cos\alpha \cos\omega t-r_{St}\sin\alpha \sin\omega t\right)\left(\begin{array}{c} \cos\alpha \\ \sin\alpha \end{array}\right) \end{array}$$

where *R* is the reflection matrix of the tested sample. The intensity of the interference signal received by D3 is:

(13) $$I_{test}={\left|E_{test}\right|}^{2}=I_{0test}\left(1+\cos\left(2\omega_{1}t+\varphi_{t}\right)\right)$$

the average intensity $I_{0test}=\left({\left|r_{Pt}\right|}^{2}\cos^{2}\alpha +{\left|r_{St}\right|}^{2}\sin^{2}\alpha \right)/2$ and the phase of the tested signal could be expressed as:

(14) $$\varphi_{t}=\tan^{-1}\left(\frac{2r_{\mathrm{Pt}}r_{\mathrm{St}}\sin\alpha \cos\alpha }{{\left|r_{Pt}\right|}^{2}\cos^{2}\alpha -{\left|r_{St}\right|}^{2}\sin^{2}\alpha }\right)$$

Finally, the phase difference of the reference signal and the tested signal is:

(15) $$∆\varphi =\varphi_{t}-\varphi_{r}=\tan^{-1}\left(\frac{2r_{\mathrm{Pt}}r_{\mathrm{St}}\sin\alpha \cos\alpha }{{\left|r_{Pt}\right|}^{2}\cos^{2}\alpha -{\left|r_{St}\right|}^{2}\sin^{2}\alpha }\right)-2\alpha$$

Consequently, the glucose solution reaction with the glucose sensor induced the change of $r_{Pt}$ and $r_{St}$, the phase difference ∆φ will be changed accordingly. On the other hand, from Equation (6), it can be seen that the values of $P_{1}$ and $P_{2}$ will affect the heterodyne frequency, but the correctness and calibration of the grating period were not necessary. Since the heterodyne light sources of the reference signal and the tested signal were the same, the heterodyne frequencies of these two signals will be the same. Therefore, the absolute value of the heterodyne frequency is not so significant when analyzing the phase difference. That is to say, the grating periods $P_{1}$ and $P_{2}$ do not need precise calibration.

## 3. Experimental Results and Discussion

### 3.1. Experiment Parameters Setup

**Figure 3:**
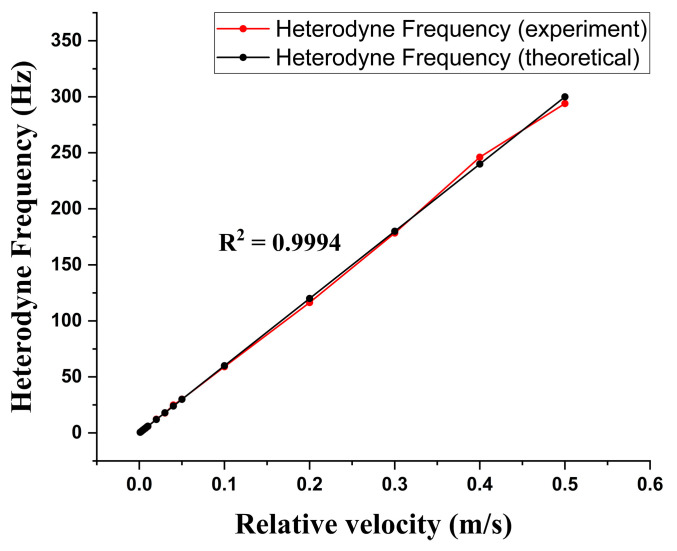
The linearity test of the heterodyne frequency versus the moving gratings’ velocities.

In this study, the wavelength of the stabilized He–Ne laser was 632.8 nm; the substrate of the sensor was made of BK7, and its refractive index was 1.5168. The period of G1 and G2 was 600 groovs/mm, and the moving velocities of the two gratings were $v_{1}=-v_{2}=0.250\;mm/s$. Hence, the predicted heterodyne frequency was equal to 300 Hz. To show the validity of the moving-grating-based heterodyne light source, we tested the linearity of the heterodyne frequency versus the moving gratings’ velocities, and the result is shown in Figure 3.

The linearity index could be calculated by the R-square of the experimental data set and was equal to 0.9994. As per our expectation, the heterodyne frequency is linearly proportional to the relative velocity of the two gratings. We can still observe some frequency difference between experimental results and theoretical prediction, and the maximum difference could be around 2%. This frequency difference is due to the accuracy of the velocity of the moving gratings. Based on this result, we can test and control the accuracy of the velocity of a moving grating.

**Figure 4:**
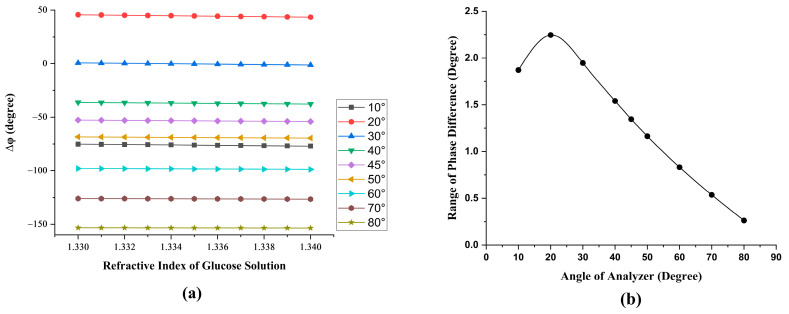
(**a**) The simulated phase change under different angles of the analyzer. (**b**) The range of the phase change versus different angles of the analyzer.

The refractive index of the glucose solution was about 1.33 to 1.34. The grating G3 used for deflection light had a period of 1200 groovs/mm. According to the diffraction formula [20], the diffractive angle of the positive first-order diffracted light was 49.4°. Therefore, the incident angle θ=49.4°. According to Equation (15), the measured phase difference is related to the transmission axis angle (α) of the AN2 and AN3, and the simulation result of the phase change can be obtained. As shown in Figure 4a, the phase difference is nearly linearly proportional to the refractive index in the range of 1.33 to 1.34. Therefore, the best transmission axis angle of AN2 and AN3 can be determined by the range of phase change, as shown in Figure 4b. α=20° was chosen for the transmission axis angle of the AN2 and AN3 in the experiment.

### 3.2. The Glucose Oxidase Coating on the Sensor

**Figure 5:**
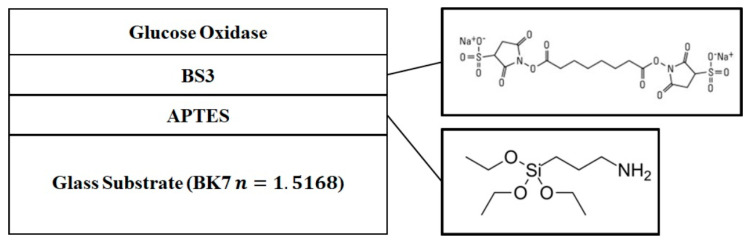
The glucose concentration sensor structure.

In order to control the accuracy of the experiment, a glucose oxidase film was deposited on the sensor. However, since glucose oxidase is not easily deposited on the substrate directly, it needs multi-chemicals as a medium [21]. The completed sensor structure is shown in Figure 5. The first layer above the glass substrate is trimethoxysilane (APTES), where the branched chain containing the Si–O structure can be bonded to the glass substrate. The second layer above the glass substrate is bis(sulfosuccinimidyl)suberate (BS3), which is used for cross-linking agents between APTES and glucose oxidase. Finally, glucose oxidase can be stably deposited on the glass substrate of the sensor. 

### 3.3. Tests of the Validation Period of the Glucose Sensor

**Figure 6:**
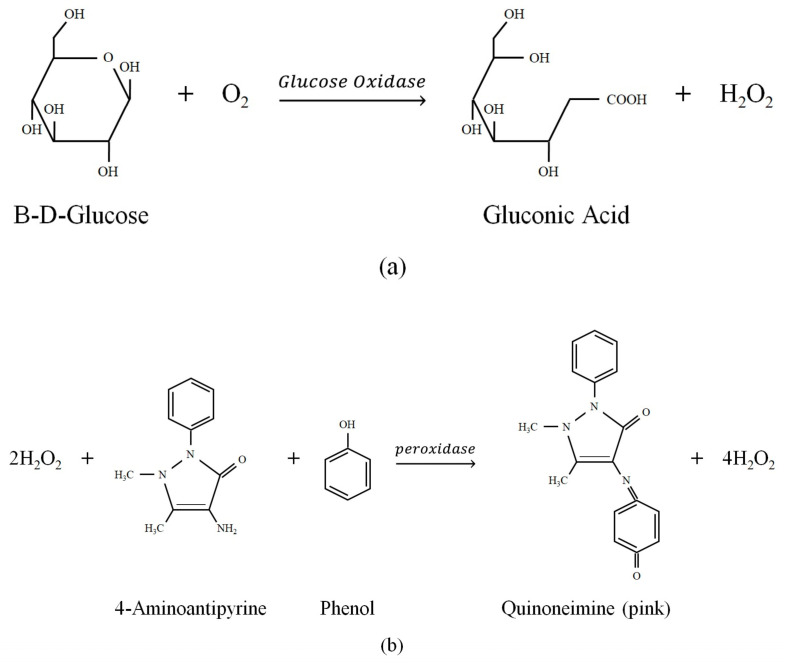
(**a**) The reaction between the glucose and the Glucose Oxidase (GOx). (**b**) The reaction between the detection solution and the H_2_O_2_.

The glucose oxidase could react with glucose; the reaction chemistry formula is shown in Figure 6. After the reaction between the glucose and the Glucose Oxidase (GOx) was deposited on the sensor, as shown in Figure 6a, gluconic acid and hydrogen peroxide (H_2_O_2_) were produced. The colorimetric method provided by the WHO [22] was used to ensure that the GOx was indeed bonded to the glass surface and whether there was an active reaction. The detection solution used in the colorimetric method contains 4-Aminoantipyrine, Phenol, and Peroxidase. After the detection solution reacted with the H_2_O_2_, it produced Quinoneimine, and the complete chemical reaction formula is shown in Figure 6b [21]. After the reaction is complete, it can be judged whether the sensor is still active according to the degree of pink of the solution. From the reaction equation, the degree of pink of the reaction product is related to glucose concentration in the solution. The higher the glucose concentration, the higher the Quinoneimine, and the darker the pink. Moreover, after different concentrations of glucose solutions react with the sensor, the refractive index of the glucose solution will be changed. Therefore, it can be known from Equation (15) that when the refractive index changes, the phase of the interference signal will change accordingly. Therefore, we call this sensor a glucose concentration sensor.

**Figure 7:**
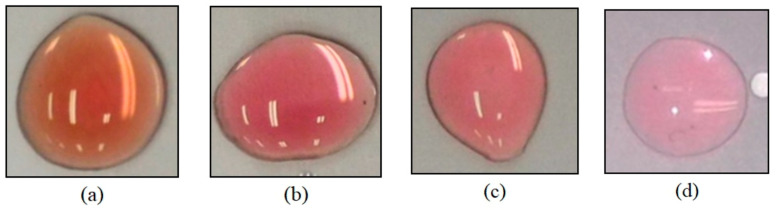
The colorimetric results of glucose sensor test strips stored for (**a**) 0 days, (**b**) 30 days, (**c**) 60 days, and (**d**) 90 days with a 200 mg/dL glucose solution.

In order to understand the impact of the storage time on the glucose sensor, four different storage times were tested for validating storage periods under a controlled environment where the temperature and relative humidity were 25°±0.1° and 45±2.5%. Compared with the color of glucose sensor test strips stored for 0 days, 30 days, 60 days, and 90 days with a 200 mg/dL glucose solution, the results are shown in Figure 7. After 30 days of storage, the colorimetric results still maintained a certain degree of dark pink. After 60 to 90 days, the colorimetric results showed a lighter pink color. The activity of the GOx coating was significantly reduced. Therefore, the validation storage period of the glucose sensor made in this research was about 30 days.

**Figure 8:**
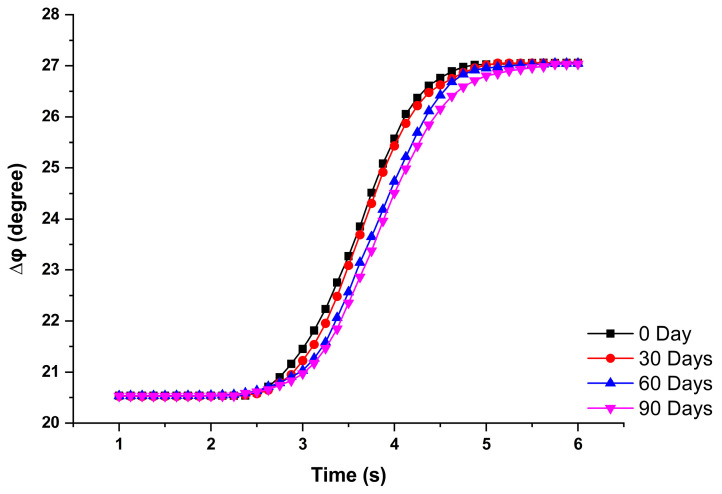
The phase change measurement of different preservation times.

To identify the validation storage period more accurately and by quantization methodology, we tested the reaction rate. The sensor’s preservation time affects the accuracy of the measurement and the rate of chemical reactions. As shown in Figure 8, there were four curves with different preservation times. 

In Figure 8, the y-axis represents the phase change of the interference signal, while the x-axis represents the time elapsed during the experiment. Initially, the interference signal phase was maintained at a lower level of approximately 20.5°. Following the titration of the glucose solution onto the sensor, the reaction commenced, resulting in a gradual increase in the phase change. Eventually, upon completion of the reaction, the interference signal phase was maintained at a higher level of approximately 27° during the final stage of the experiment. Hence, we set the 10% and 90% of the phase change value as the reference for the beginning and end of the reaction; that is, when the phase reaches 21.1841° and 26.4019°, the reaction is regarded as the beginning and end of the reaction, respectively. Furthermore, the difference of the 10% and 90% of the phase changed values represents the phase change value due to the reaction. The comparison of the storage time and phase change rate is shown in Table 1. After the glucose sensor was stored for 90 days, its reaction time increased by 1.25 s, and the reaction rate decreased from 3.7947 deg/s to 1.9877 deg/s, a decrease of 47.6%. On the other hand, for the 30 days validation period defined by the color rendering method described in the previous paragraph, the reaction rate decreased from 3.7947 deg/s to 3.2109 deg/s, with a difference of 15.34%. Compared with the colorimetric method, the phase change detection method used in this study can monitor the changes in the reaction process and quantify the reaction rate accurately.

### 3.4. Measurement Results of Different Glucose Concentrations

**Figure 9:**
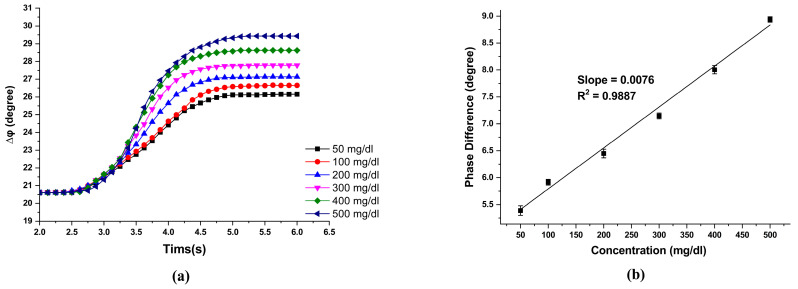
(**a**) The simulated phase change under different angles of the analyzer; (**b**) The range of the phase change versus different angles of the analyzer.

The concentrations of glucose solution used in this study were 50 mg/dL, 100 mg/dL, 200 mg/dL, 300 mg/dL, 400 mg/dL, 500 mg/dL, and 40 μL. After titrating the solution, the reaction was completed within 6 s, and the phase value was stable. Figure 9a shows the overall phase change value of about 5°~8° after the reaction was completed. We repeated ten sets of experiments for each concentration of glucose solution. The phase change difference values of different concentrations of the glucose solution and the associated fitting curve are shown in Figure 9b. The slope of the fitting curve 0.0076 deg/(mg/dL) represents the sensitivity of the phase change of the measurement system to the glucose concentration, and the R-square value was calculated to be 0.9887. 

**Figure 10:**
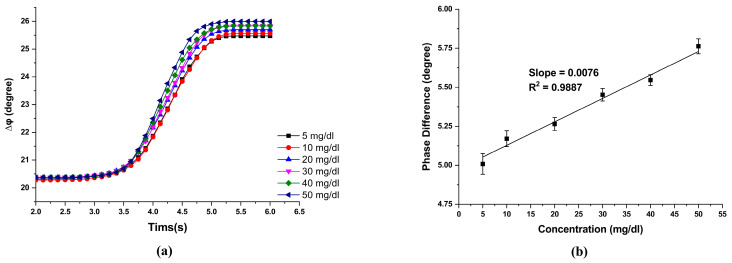
Measurement results of low-concentration glucose solution. (**a**) Reaction curves of different concentrations of glucose solution. (**b**) The phase change of different concentrations of glucose solution and the associated fitting curve.

However, generally speaking, the difference between the highest and lowest blood glucose values in humans is about 40 mg/dL. The measurement system must have a high enough resolution to measure accurately at low concentrations. Six low concentrations of 5 mg/dL, 10 mg/dL, 20 mg/dL, 30 mg/dL, 40 mg/dL, and 50 mg/dL were used to verify the measurement accuracy at low concentrations. The phase difference results of different glucose concentrations are shown in Figure 10a, and the associated fitting curve is shown in Figure 10b. The slope of the calibration line was 0.0150 deg/(mg/dL), and the R-square value was 0.9736. From this result, it can be seen that the lower the concentration, the higher the measurement sensitivity.

### 3.5. Measurement Resolution of this Methodology

Next, the phase difference measurement mainly came from the error of incident angle $\delta \varphi_{\theta }$, polarization mixing error $\delta \varphi_{m}$, second harmonic error $\delta \varphi_{R}$, the error of the polarization transmission axis angle $\delta \varphi_{\alpha }$, and the resolution of the lock-in amplified $\delta \varphi_{lock}$. The error $\left|\delta \varphi_{\theta }\right|$ came from the misalignment of the incident angle. In our experiment, it relates to the resolution of the rotation stage and was about 0.1°. Hence, the corresponding $\left|\delta \varphi_{\theta }\right|=0.007{}^{\circ}$. Next, owing to the extinction ratio effect of a polarizer, the mixing of light polarization occurs. $\left|\delta \varphi_{m}\right|$ can be reduced to 0.008° as the extinction ratio of the AN is $1\times 10^{-4}$. The errors $\delta \varphi_{R}$ and $\delta \varphi_{\alpha }$ were related to the resolution of the rotation stage used for the polarizer and analyzer, and it was about 0.1°, so we can calculate $\left|\delta \varphi_{R}\right|=0.0001{}^{\circ}$ and $\left|\delta \varphi_{\alpha }\right|=0.0120{}^{\circ}$. The phase-detection error in our experiment was related to the resolution of the lock-in amplifier used in our experiment, and it was 0.001°. Consequently, the total measured phase error can be calculated by $\delta \left(∆\varphi \right)=\delta \varphi_{\theta }+\delta \varphi_{m}+\delta \varphi_{R}+\delta \varphi_{\alpha }+\delta \varphi_{lock}=0.0281{}^{\circ}$, and the phase error was 0.2~0.3% to the measured phase (20°~30°). Based on the slope of Figure 10b, the resolution of this study was around 2 mg/dL.

## 4. Conclusions

The proposed research introduces an all-grating-based system for glucose concentration measurement, which combines a moving-grating-based heterodyne light source and a grating-based self-align sensor. The heterodyne light source was obtained by light passing through a pair of moving gratings and combining the two first-order diffraction lights by a polarization beam splitter. Then, the S- and P-polarized light interfere with an analyzer, and the interference signal is a heterodyne light source with a heterodyne frequency depending on the gratings’ moving velocities. The linearity between the gratings’ moving velocities and the heterodyne frequency calculated by the R-square equal to 0.9994. Next, to make the optical configuration setup easy and accurate, a grating-based self-align sensor was used, which was contained by two diffraction gratings to control and receive the test light beam. Moreover, the sensor was deposited on a GOx film to improve the measurement sensitivity and specificity for glucose. Finally, the phase change induced by the reaction of the sensor and glucose solutions was detected. The validity of this method was proved, and the measurement resolution can reach 2 mg/dL. Compared to the traditional EOM-based heterodyne interferometer, the advantages of the moving-grating-based heterodyne interferometer proposed in this method were cost-effectiveness, ease of use, optical setup, and wavelength limitation. On the other hand, compared with the electrochemical method, this method has an advantage in the short measurement time and long lifetime of the sensor. However, the method proposed in this study is currently only applicable to laboratory measurements and is not a portable device. In future work, a long-wavelength light source should be used to penetrate the skin to reach the vascular tissue, or sweat should be used as the tested sample for noninvasive monitoring purposes.

## Figures and Tables

**Table 1:** Comparison of the storage time and phase change rate.

Storage Time (Day)	Reaction Time (s)	Phase Change Rate (deg/s)
0	1.375	3.7974
30	1.625	3.2109
60	2.125	2.4554
90	2.625	1.9877

## Data Availability

Not applicable.
